# Structural Evolution and Transition Dynamics in Lithium Ion Battery under Fast Charging: An Operando Neutron Diffraction Investigation

**DOI:** 10.1002/advs.202102318

**Published:** 2021-09-08

**Authors:** Xianyang Wu, Bohang Song, Po‐Hsiu Chien, S. Michelle Everett, Kejie Zhao, Jue Liu, Zhijia Du

**Affiliations:** ^1^ Electrification and Energy Infrastructures Division Oak Ridge National Laboratory Oak Ridge TN 37830 USA; ^2^ School of Mechanical Engineering Purdue University West Lafayette IN 47907 USA; ^3^ Neutron Scattering Division Oak Ridge National Laboratory Oak Ridge TN 37830 USA

**Keywords:** fast charging, lithium ion batteries, Li*_x_* C_6_ phase transition, operando neutron diffraction

## Abstract

Fast charging (<15 min) of lithium‐ion batteries (LIBs) for electrical vehicles (EVs) is widely seen as the key factor that will greatly stimulate the EV markets, and its realization is mainly hindered by the sluggish diffusion of Li^+^. To have a mechanistic understanding of Li^+^ diffusion within LIBs, in this study, structural evolutions of electrodes for a Ni‐rich LiNi_0.6_Mn_0.2_Co_0.2_O_2_ (NMC622) || graphite cylindrical cell with high areal loading (2.78 mAh cm^−2^) are developed for operando neutron powder diffraction study at different charging rates. Via sequential Rietveld refinements, changes in structures of NMC622 and Li*_x_* C_6_ are obtained during moderate and fast charging (from 0.27 C to 4.4 C). NMC622 exhibits the same structural evolution regardless of C‐rates. For phase transitions of Li*_x_* C_6_, the stage I (LiC_6_) phase emerges earlier during the stepwise intercalation at a lower state of charge when charging rate is increased. It is also found that the stage II (LiC_12_) → stage I (LiC_6_) transition is the rate‐limiting step during fast charging. The LiC_12_ → LiC_6_ transition mechanism is further analyzed using the Johnson–Mehl–Avrami–Kolmogorov model. It is concluded as a diffusion‐controlled, 1D phase transition with decreasing nucleation kinetics under increasing chargingrates.

## Graphical abstract

Operando neutron diffraction has been used to study the structural evolutions and phase transitions in a LiNi_0.6_Mn_0.2_Co_0.2_O_2_ (NMC622) || graphite cylindrical cell at different charging rates. The phase transition from LiC_12_ to LiC_6_ is a limiting step under fast charging, which is a diffusion‐controlled, 1D process with decreasing nucleation kinetics via the Johnson–Mehl–Avrami–Kolmogorov model.

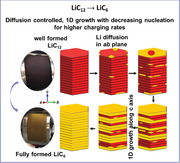

## 1 Introduction

Since its first commercialization by Sony in 1991, Li‐ion batteries (LIBs) have dominated the portableelectronic markets during the past 30 years.^[^ ^1^ ^]^ These days, the huge demand from electrical vehicles (EVs) market challenges the state‐of‐the‐art LIB technologies for higher energy density and shorter charging time to less than 15 min,^[^ ^2^ ^]^ to ease the range anxiety and promote the adoption of LIBs in EV markets. Utilization of realistic loading electrodes (3–4 mAh cm^−2^) has been well proven as an effective way of increasing the cell energy density.^[^ ^3^ ^]^ However, the caveat is that thicker electrodes prevent the cell from fast charging, which can lead to unwanted Li plating and eventual cell failure.^[^ ^4^ ^]^ It is generally recognized that the limiting factor of fast charging is the limited transport property of Li^+^ in electrolytes and/or graphite anode. Under fast charging (high C rate) conditions, Li^+^ concentration gradient builds up in both the electrolyte and graphite, leading to insufficient lithiation of graphite and Li plating on graphite electrode. Several approaches have been reported to improve the fast charging performance of LIBs via improving Li^+^ mass transport at the graphite electrode side, including advanced electrolyte formulation, asymmetric temperature modulation, and laser‐patterned electrode architectures.^[^ ^5^ ^]^ Anode overpotential control via interfacial modification has also been reported to suppress Li plating during fast charging.^[^ ^6^ ^]^ While these studies shed light on improvements of fast charging, the structural evolution of electrode materials (both the layered cathode and anode materials) during fast charging has not been systematically studied. Further investigation, especially under operando conditions, is highly needed.

For fast charging of LIBs, as the challenge comes mainly from the Li^+^ diffusion/intercalation at the graphite anode side in a nonequilibrium fashion, the intercalation of Li^+^ into graphite via staging has been intensively investigated.^[^ ^7^ ^]^ From the domain model proposed by Daumas and Herald to the Cahn–Hilliard type phase‐field model, they provide solid mechanistic understanding and explanation of the staging transition during lithium intercalation.^[^ ^8^ ^]^ Valuable insights on the staging process have been elaborated from various characterization methods. Electrochemical methods (EIS, PITT, and GITT) were reported to obtain valuable information on the kinetic behaviors of the Li intercalation.^[^ ^9^ ^]^ Semi‐quantitative studies on the Li^+^ transport within graphite were reported by observing the color changes for lithiated graphite at different states of charges via optical imaging.^[^ ^10^ ^]^ The qualitative structural evolutions and stagings during intercalation were also widely probed via in situ/ex situ Raman spectra;^[^ ^11^ ^]^ other experimental methods such as NMR, in situ X‐ray/synchrotron and neutron powder diffraction (NPD) also provide insightful qualitative information on the Li^+^ intercalation.^[^ ^12^ ^]^ However, detailed quantitative information about the structural evolution of graphite staging phases, especially under fast charging conditions, is still lacking.

X‐ray and neutron powder diffraction techniques have been well developed due to their unique capability on structural characterization.^[^ ^13^ ^]^ For applications of in situ/operando X‐ray powder diffraction on LIBs under fast charging, encouraging progresses have been made recently from two groups via high speed synchrotron diffraction with spatial resolution at micron scale on thick graphite anodes. Koffi P. C. Yao et al. quantified the Li^+^ concentration gradient and staging kinetics within a 114 µm thick graphite anode under up to 1 C charging.^[^ ^14^ ^]^ Donal P. Finegan et al. obtained temporal and spatial distribution of graphite intercalation compounds (GICs) and plated Li metal for a 101 µm thick graphite anode under up to 6 C charging.^[^ ^15^ ^]^ Then they quantitatively analyzed the kinetics of phase transition and the heterogeneities of Li plating. For NPD techniques, in situ NPD has been applied to the study of LIBs ever since early 2000.^[^ ^16^ ^]^ Compared to the widely used synchrotron X‐ray diffraction techniques for the structural characterization of LIBs, NPD has the following unique advantages: 1) strong penetration and no heating effect; the heating effect from high energy photons often leads to inhomogeneous or delayed electrochemical reactions within LIBs, despite improved signal‐to‐noise ratio as a result of the very high flux photons; 2) neutron scattering lengths are constant as a function of momentum transfer (*Q*), which helps to better quantify the site occupancies and atomic displacements; 3) NPD can detect light elements in the electrode materials, especially carbon and oxygen, which play a pivotal role in the structural evolution for LiNi_1−_ *_x_*_−_ *_y_* Mn*_x_* Co*_y_* O_2_ (NMC) cathode materials and graphite anodes; 4) NPD can distinguish adjacent transition metal ions such as Ni, Co, and Mn, which is an otherwise challenging task to cope with when using conventional X‐ray diffraction.^[^ ^17^ ^]^ Despite these great advantages, the broad application of NPD has been hindered by the relatively lower neutron flux and the larger amount of materials needed. Also, it suffers from large incoherent scattering from hydrogen‐rich organic electrolyte, and scarce neutron beam time.^[^ ^13b^ ^]^

Despite the above challenges, steady progress has been made in improving the capability of in situ/operando neutron diffraction in the past decade. For instance, Neeraj Sharma et al. investigated the phase transition of Li*_x_* C_6_ via operando NPD study by overcharging a commercial 18 650 cell to ≈4.6 V.^[^ ^18^ ^]^ Lucien Boulet‐Roblin et al. studied a LiNi_0.5_Mn_1.5_O_4_ (LNMO) || graphite cylindrical cell filled with deuterated electrolyte, and they were able to reveal the evolution of lattice parameters of both the LNMO and graphite phases.^[^ ^13e^ ^]^ Laura Vitoux et al. designed a LiNi_0.6_Mn_0.2_Co_0.2_O_2_ (NMC622) || Li cylindrical cell with the capability of repeated cycling up to 60 cycles, and the signal‐to‐noise ratio was good enough to extract the structure information of NMC622.^[^ ^19^ ^]^ Jörn Wilhelm et al. probed the low temperature operation of a pouch cell under various discharging rates and following relaxation via in situ NPD, and by studying the co‐existence of LiC_12_ and LiC_18_ phases, they elaborated the inhomogeneous lithiation within the graphite anode at low temperature.^[^ ^20^ ^]^ Yet, collecting structural refinable operando neutron diffraction data under fast charging conditions is still extremely challenging, in large part due to the relatively long data acquisition time required at many neutron powder diffractometers.^[^ ^21^ ^]^ Recently, we successfully commissioned the operando neutron diffraction study of batteries at the Spallation Neutron Source (SNS)’s NOMAD beamline. The high incident neutron flux together with very large detector coverage at NOMAD enables the very fast collection (<1 min per pattern) of high quality operando neutron diffraction data from the newly designed cylindrical cell. This inspires us to carry out a systematic investigation of the structural evolution of NMC622 and graphite electrodes during fast charging.

All the information obtained (lattice structure, phase ratio, and their time evolution) is vitally important for advancing the fast charging technologies of LIBs. Thus, the operando neutron diffraction capability developed by our group offers a unique opportunity to address those aforementioned issues/challenges. In this study, a customized cylindrical cell of NMC622 || graphite was used for fast operando neutron diffraction under various charging rates from 0.27 C to 4.4 C. Structural changes in both NMC622 and graphite are obtained and analyzed using sequential Rietveld refinements. The phase transition from stage II (LiC_12_) to stage I (LiC_6_) is demonstrated to be the rate‐limiting step during Li intercalation into graphite under fast charging conditions. The dynamics of LiC_12_ → LiC_6_ transition is further investigated via the classical Johnson–Mehl–Avrami–Kolmogorov (JMAK) model, which is found to follow a 1D diffusion‐controlled growth mechanism. These findings provide fundamental insights for further optimizing graphite‐based anode for fast charging LIBs.

## 2 Results and Discussion

**Figure 1:**
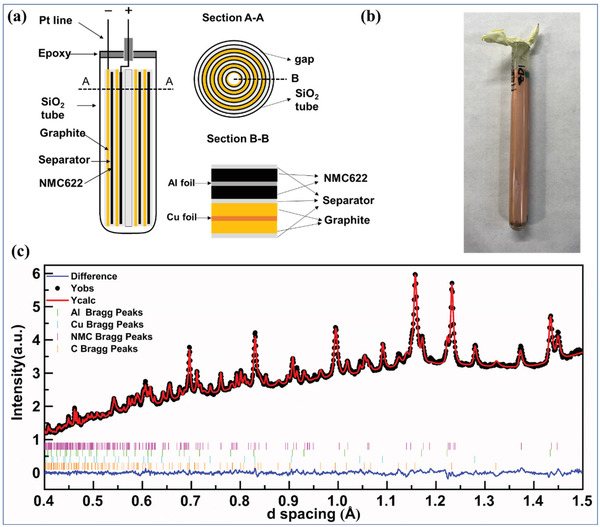
Experimental set‐up for the operando neutron diffraction experiments: a) schematic illustration of the cylindrical cell; b) a typical cylindrical cell utilized in the operando experiment; c) Rietveld refinement on collected NPD data.

The NMC622 || graphite cylindrical cell was prepared at the DOE Battery Manufacturing R&D Facility (BMF) at Oak Ridge National Laboratory. The NMC622 electrode consisted of 3 wt % carbon black (Denka Li‐100), 3 wt % polyvinylidene fluoride (PVDF, Solvay 5130), and 94 wt % NMC622 (Targray). The areal capacity was 2.78 mAh cm^−2^ with 30% porosity after calendaring. The thickness of NMC622 cathode and graphite anode was 57 and 59 µm, respectively. The graphite electrode consisted of 5 wt% of PVDF (KUREHA KF9300), 1 wt% of carbon black (Denka Li‐100), and 94 wt% of graphite (Superior graphite, 1506T). The negative/positive (N/P) ratio was set at 1.1. The jelly roll was prepared by sandwiching the cathode and anode with the separator (Celgard 2325). A thin‐walled quartz tube was used to contain the jelly roll, which was filled with nonaqueous electrolyte (BASF, 1.2 M/L LiPF_6_ EC/EMC with 30:70 wt%). The design for a typical cylindrical cell was schematically illustrated in **Figure** **1**a and the cell prepared for this work was shown in Figure 1b. The cell was cycled between 2.8 and 4.2 V versus Li^+^/Li using a VMP‐300 potentiostat (Biologic) with constant current constant voltage (CCCV) charging protocol from low to high charging rates: 0.27 C, 1.6 C, 2.4 C, 3.2 C, and 4.4 C. The detailed charging/discharging curves were shown in Figures S1 and S2, Supporting Information.

The operando NPD experiments were carried out at the SNS's NOMAD beamline, and the cell was charged/discharged at 20 °C. The NPD data was collected in operando mode (no relaxation period). Due to the relatively lower (neutron) flux at NOMAD (which is already one of the highest fluxed neutron diffractometers worldwide) compared to the synchrotron X‐ray diffractometers, the required data collection time in this operando study is relatively longer than that using synchrotron X‐ray diffraction (seconds or less). To achieve good signal‐to‐noise ratio of operando diffraction data that allows accurate structure refinements, a minimum of ≈1 min data collection time is needed, thus the data collection time was set as 62 s for the operando experiments.

The extracted information on staging kinetics is spatial averaged, due to the relatively large neutron beam size (≈1 × 1 cm^2^) and strong penetration of neutron beam. Therefore, information on spatial distribution of various GIC phases is lost. In addition, the spatial and temporal distributions of Li^+^ within electrodes keep evolving during the data collection, and the Bragg peaks (intensity and peak position) from each phase keep changing due to evolving Li^+^ contents within each phase. Therefore, the collected diffraction data is a superposition of these time evolving peaks from phases with different SOC, which is a time average result.

During the operando experiments, time‐of‐flight (TOF) are converted to *d*‐spacing using the conventional second order polynomial TOF = *ZERO* + *DIFC* × *d* + *DIFA* × *d^2^*, where *ZERO* is a constant, *DIFC* is the diffractometer constant and *DIFA* is an empirical term to correct for sample displacements and absorption induced peak shifts. *ZERO* and *DIFC* were determined from the refinement of a standard NIST Si‐640e data set and held fixed. The same *DIFA* value was adopted for all sequential refinements since the cylindrical cell was not moved during the entire experiment. For the low‐resolution frames (bank 2 and 3), a back to back exponential function convoluted with a symmetrical Gaussian function was used to describe the peak profile. For the high‐resolution frames (bank 4 and 5), the moderator induced line profile was modeled using a modified Ikeda‐Carpenter‐David function.^[^ ^22^ ^]^ Lorenz polarization is corrected by multiplying *d^4^Sinθ*.^[^ ^22b^ ^]^ Absorption correction was carried out using the empirical Lobanov formula.^[^ ^22^, ^23^ ^]^ TOPAS Academic V6 suite was utilized to perform the sequential Rietveld refinements on all diffraction patterns obtained during the charging/discharging process.^[^ ^24^ ^]^ The weightings of Cu and Al were fixed to values refined from the initial patterns (before charging). The cation mixing extent and atomic displacements for the cathode were fixed during the sequential refinement. The Li content within NMC622 was fixed to the value from the electrochemical data. The lattice parameters of pristine NMC622 and Li‐intercalated graphite are thus obtained from refinement using ex situ diffraction data of pristine powders. The refinement result of one operando diffraction pattern is shown in Figure 1c.

**Figure 2:**
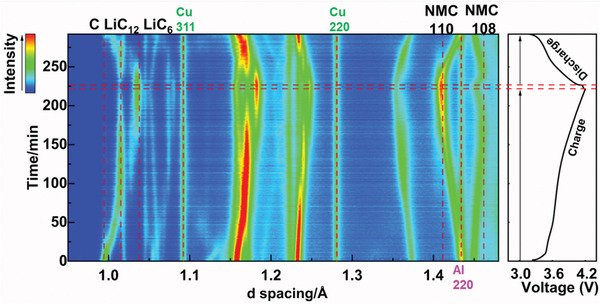
The structure/phase evolution of graphite and NMC622 under 0.27 C charge and 1 C discharge.

**Figure 3:**
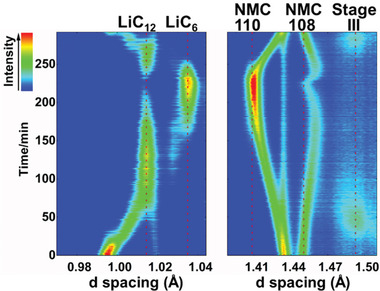
The formation of stage III (LiC_30_), stage II (LiC_12_), and stage I (LiC_6_) under 0.27 C charge and 1C discharge.

The structural evolution of graphite anode and NMC622 cathode are first studied at relatively slow charging rate to demonstrate the feasibility and reliability of this cylindrical cell design. The contour plots of the operando diffraction data alongside the voltage profile of the cylindrical cell under 0.27 C charging and 1 C discharging are shown in **Figure** **2**. The peak shifting, intensity variation, and appearance/disappearance of certain Bragg peaks clearly suggest the continuous structural changes of both NMC622 and GICs during lithium intercalation/de‐intercalation. As shown in the enlarged contour plot (**Figure** **3**), the intercalation of Li^+^ into graphite undergoes a sequential staging process: from the initial solid solution process (pristine graphite) to stage III (LiC_30_), dilute stage II (LiC_18_), stage II (LiC_12_) and the final stage I (LiC_6_), agreeing with previous studies.^[^ ^25^ ^]^ This sequential staging process is mainly a response to reducing the elastic and electrostatic interactions of inserted Li ions between graphene layers.^[^ ^26^ ^]^ Detailed phase transition process during Li intercalation (charging process) are summarized in Figure S3, Supporting Information. Upon initial intercalation of Li^+^, no new Bragg peaks are detected and the graphite (114) reflection (S.G. *P*6_3_ */mmc*) keeps shifting to larger *d*‐spacing, indicating the initial Li^+^ intercalation follows a solid solution reaction. When ≈0.15 Li^+^ (Li_0.15_C_6_) are intercalated, the stage III of GICs (LiC_30_ with A(Li)ABA… stacking, S.G. *P*3) phase starts to emerge, as indicated by the appearance of a broad peak at ≈1.49 Å (Figure 3, which corresponds to the 007 reflection). Further intercalation of Li^+^ leads to the formation of the dilute stage II phase (LiC_18_ with AB(Li)BA··· stacking, S.G. *P*6_3_ *mc*) and then the stage II phase (LiC_12_ with AA(Li)AA··· stacking, S.G. *P*6/*mmm*). The difference between LiC_18_ and LiC_12_ is the detailed stacking sequences of neighboring graphene layers between adjacent Li layers: the former follows the AB type registration while the latter follows the AA type stacking.^[^ ^27^ ^]^ The rapid increase of the (002) reflections of LiC_12_ (≈1.02 Å) indicates that all LiC_18_ from dilute stage II have eventually been transformed into stage II (LiC_12_). With more Li^+^ intercalation, the stage II (LiC_12_) starts to gradually transform into the fully lithiated stage I (LiC_6_) with a new set of (002) reflections (S.G. *P*6/*mmm*) at larger *d*‐spacing of ≈1.04 Å. A small amount of LiC_12_ still coexists with LiC_6_ at the end of charging mainly due to the use of extra amounts of graphite (e.g., the N/P ratio of 1.1). During discharge, the phase transition is largely reversible (Figures 2 and 3).

**Figure 4:**
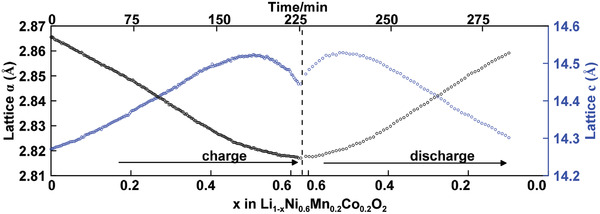
The lattice parameters *a* and *c* evolution of NMC622 under the 0.27 C charging and 1 C discharging.

For the cathode side, the de‐intercalation of Li^+^ from NMC622 leads to the oxidation of Ni^2+^/Ni^3+^, which compensates the charge loss caused by Li^+^ removal.^[^ ^28^ ^]^ The oxidation of transition metal (TM) cations decreases their ionic radii and increases the covalency between TM cations (e.g., Ni and Co) and ligand oxygen anion, leading to the decrease of average TM—O bond lengths and the subsequent decrease of lattice parameter *a* (from ≈2.865 Å to ≈2.817 Å, **Figure** **4**). This is clearly indicated by the shift of the 110 reflection toward lower *d*‐spacing (Figure 3), which changes from the initial ≈1.43 Å to ≈1.41 Å. Lattice parameter *c* first increases to its maximum value of ≈14.52 Å (at *x* = ≈0.5 in Li_1−_ *_x_* Ni_0.6_Mn_0.2_Co_0.2_O_2_), and then decreases to ≈14.45 Å (*x* = ≈0.622) at the end of charging (Figure 4). This trend is consistent with the observation of the shift of 108 reflection (Figure 3), which first increases from ≈1.44 Å to ≈1.45 Å and then decreases to ≈1.44 Å. The de‐intercalation of Li^+^, on one hand, effectively reduces the screening effect between adjacent oxygen planes, leading to the increase of electrostatic repulsion between oxygen planes (across the LiO_2_ slab) and thus causes the increase of lattice *c*. On the other hand, the increase of the TM—O bond covalency results in the charge redistribution between TM cation and oxygen anion, leading to the partial charge depletion of ligand oxygen anion. This effectively reduces the electrostatic repulsion between oxygen anions across the inter‐layer plane and leads to the decrease of lattice *c*. The change of lattice *c* is determined by the relative strengths of these two effects.^[^ ^17^, ^29^ ^]^ The changes in lattice parameters *a* and *c* are in good agreement with previous studies on NMC cathode materials.^[^ ^30^ ^]^ During cell discharging, the lattice parameters *a* and *c* follow the opposite trend of the charging process (Figure 4), consistent with the peak shifting in Figure 3. Due to the irreversible loss of Li ions from the formation of SEI and kinetic hindrance, lattice parameters *a* and *c* do not fully recover to their initial values.^[^ ^31^ ^]^

**Figure 5:**
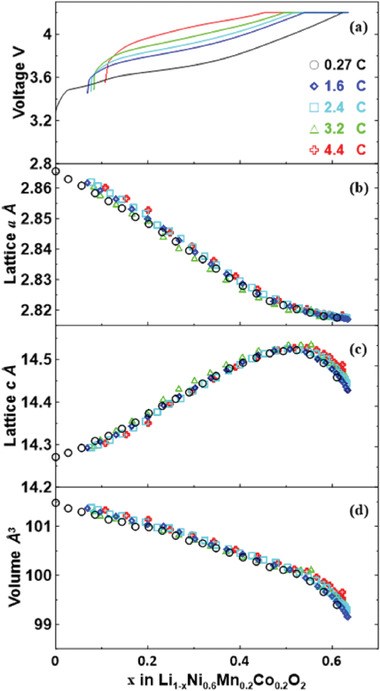
Lattice parameter evolution for NMC622 under different charging rates: a) The evolution of charging voltage during charging; b) The lattice parameter *a*; c) The lattice parameter *c*; d) The volume shrinkage during charging process.

Quantitative analysis was then carried out to better understand the structural evolution of NMC622 cathode and graphite anode at higher charging rates. **Figure** **5** shows the variation of lattice parameters of NMC622 under different charging rates up to 4.4 C, which has similar trend compared to Figure 4. This indicates the crystal structure of NMC622 is well maintained during fast charging, which is consistent with previous reports on the capability of NMC to be intercalated/de‐intercalated up to 10 C.^[^ ^32^ ^]^ Small differences can be seen in lattice parameters under different charging rates, which is attributed to the averaging effect of powder diffraction analysis and larger Li concentration gradient within NMC622 under higher charging rate. During the charging process, Li ions de‐intercalate from the surface layer of NMC622 particles first and a Li ion concentration gradient builds up from the surface to the core of the particle. Higher charging rate leads to greater concentration gradient, and the diffraction analysis is based upon the averaged data collected in 62 s and thus shows small variations.^[^ ^33^ ^]^

**Figure 6:**
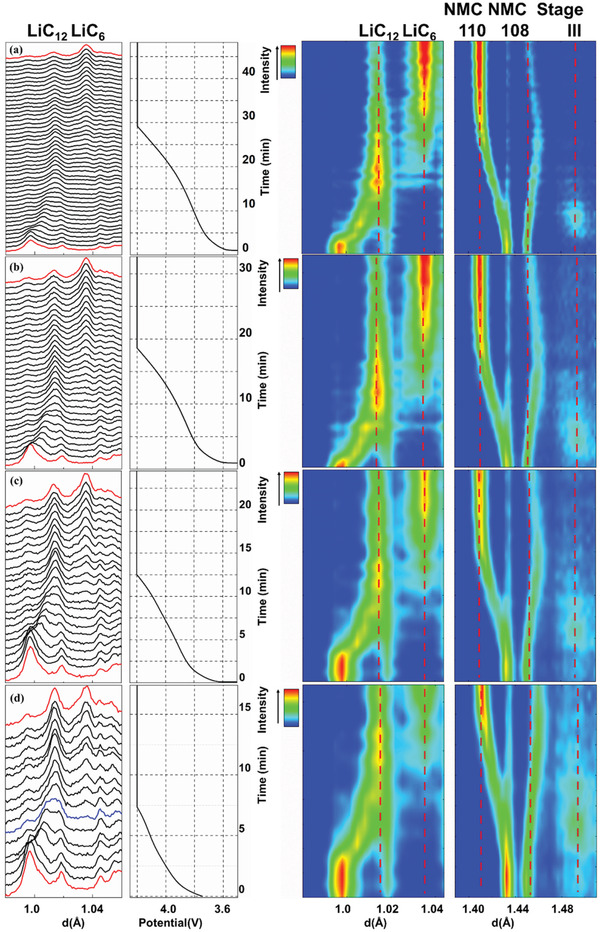
The phase evolution of graphite anode during the charging process under different charging rates: a) 1.6 C; b) 2.4 C; c) 3.2 C; d) 4.4 C.

For the anode side, the full diffraction patterns for the charging rates from 0.27 C to 4.4 C together with voltage profiles are shown in Figure S4, Supporting Information, and corresponding contour plots for the whole charging/discharging processes are shown in Figure S5, Supporting Information. The enlarged regions in **Figure** **6** (0.98 Å to 1.06 Å and 1.40 Å to 1.52 Å) are utilized to elaborate the detailed phase transition of graphite under these rates (≈1.6–4.4 C). The Li intercalation follows the same sequential staging as revealed in Figure 3 (slow charging at 0.27 C): from the emerging of (007) peak of LiC_30_ (stage III) at ≈1.49 Å, to the (002) peak of LiC_12_ (stage II), and finally to the (002) peak of LiC_6_ (stage I) at ≈1.04 Å.^[^ ^13^, ^34^ ^]^

For the 1.6 C and 2.4 C charging shown in Figure 6, LiC_12_ peak intensity increases to its maximum, and then starts to decrease with the increase of peak intensity of LiC_6_. When the charging rate is increased to 3.2 C and 4.4 C, however, the peak of LiC_6_ emerges and increases in intensity before LiC_12_ peak increases into its maximum intensity. Therefore, a simultaneous intensity increase of both LiC_12_ and LiC_6_ reflections (for example, the seventh diffraction pattern of 4.4 C) exists under higher charging rate. Numerical simulations by Dees et al. via electrochemical models presented the evolution of phase fraction in graphite anode at C/5, 1 C, and 4 C charging rates, and revealed that the monotonical increase of volume fraction for both LiC_12_ and LiC_6_ phases only existed under 4 C charging, similar to our observation.^[^ ^35^ ^]^ Quantitative analysis by Koffi P. C. Yao et al. via operando energy dispersive X‐ray diffraction showed more detailed evolution of simultaneous increase of both LiC_12_ and LiC_6_ under 1 C charging rate (with a 114 µm thick graphite anode). This phenomenon may be related to the inhomogeneous distribution of Li ions in the graphite electrode.^[^ ^14^ ^]^ Figure S6, Supporting Information shows the SOC of the cell when LiC_6_ phase emerges in the diffraction patterns (the uncertainty is from the capacity increment during neutron data collection period, 62 s in this case). A clear distinction can be noticed between 2.4 and 3.2 C, where LiC_6_ shows up at a relatively lower SOC under 3.2 C. This phenomenon is supported by other reports on high rate charging of Li‐ion cells. Subsequently, LiC_6_ starts to evolve from LiC_12_ in graphite electrode near separator while LiC_12_ forms at the expense of LiC_18_ (dilute stage II) in graphite electrode near current collector.

**Figure 7:**
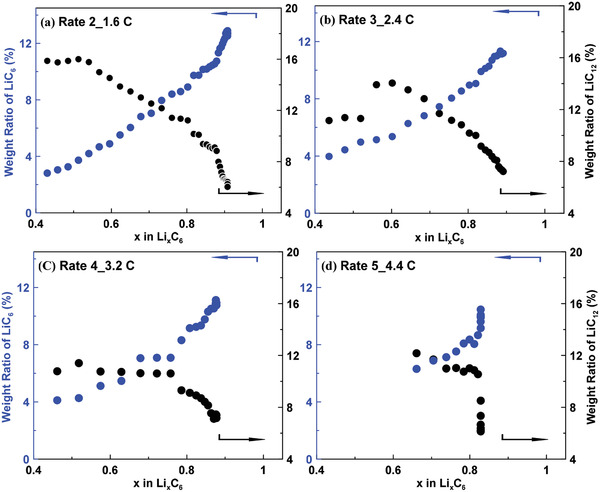
The time evolution of LiC_12_ and LiC_6_ for the whole charging process under the charging rate of a) 1.6 C, b) 2.4 C, c) 3.2 C, d) 4.4 C.

**Figure 8:**
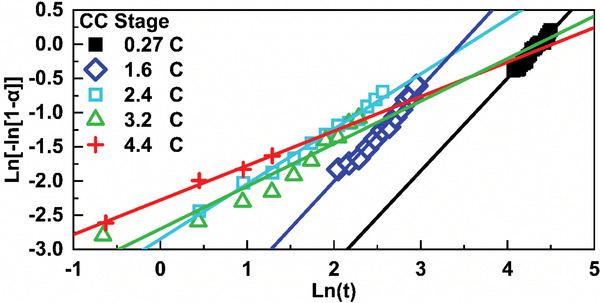
The Sharp–Hancock plot for LiC_6_ under 5 charging rates for the CC stage.

From the qualitative discussion above, it is shown that the LiC_12_/LiC_6_ two‐phase reaction is slower than the solid solution reaction from C to LiC_12_. It is therefore important to obtain the kinetics of the two‐phase reaction between LiC_12_ and LiC_6_. The relative weight ratio of LiC_6_ and LiC_12_ can be obtained qunatitatively by integrating the area of their respective diffraction peaks shown in Figure S7, Supporting Information. **Figure** **7** summarizes the time evolution of weight ratios (obtained from Rietveld refinement) of LiC_6_ and LiC_12_ at 4 different charging rates (the ratios of LiC_6_ and LiC_12_ under 0.27 C is included in Figure S8, Supporting Information). Under a given charging rate, the weight ratio of LiC_6_ keeps increasing while the weight ratio of LiC_12_ continues decreasing, which corresponds to the formation of LiC_6_ at the consumption of LiC_12_. The JMAK model, expressed in Equation (1), is then utilized to obtain more insight on the phase transition mechanism from LiC_12_ to LiC_6_. The validity of JMAK model has been well proven in previous studies to probe the dynamics of phase transition in the solid reactions of electrode materials like LiFePO_4_ and graphite.^[^ ^36^ ^]^ The Avrami exponents *n* of the JMAK model are extracted from the Sharp–Hancock plots and shown in **Figure** **8**.

(1) $${\left[-\left[\ln\left(1-a\right)\right]\right]}^{1/n}=\;\mathrm{kt}$$

For the JMAK model, the nucleation‐growth mechanism is utilized to describe the LiC_12_ → LiC_6_ transition, and it is debatable about its validity in describing this transition under high charging rates based on its inability to accurately capture the phase separation under nonequilibrium conditions. Combining direct optical images of a single crystal graphite anode under lithiation, Guo et al. utilized the Car–Hilliard reaction model to probe the lithiation of single crystal graphite.^[^ ^10c^ ^]^ Simulation results from this model well captured major lithiated phases (stage I, stage II, and stage III) which were supported by the experimental results from direct optical images. Furthermore, a multilayer free energy phase field model was developed by Smith et al. to describe the intercalation kinetics for these layer structure chemicals, especially the phase separation within them.^[^ ^37^ ^]^ However, to further confirm the proposed models (JMAK or Car–Hilliard) from atomistic structure changes, operando pair distribution function experiments (which not only probes the average phase transition but also the local/short‐range structure changes) on the LiC_12_ → LiC_6_ transition of graphite anode are needed. This will be investigated in our future work to further verify the validity of JMAK model under higher charging rates.

(2) n=a+bc

In Equation (2), the Avrami exponent *n* is divided into three components: the nucleation rate *a*, growth dimensionality *b*, and growth parameters *c*.^[^ ^38^ ^]^ Parameter *a* reveals the detailed nucleation rate: *a* = 0 corresponds for a nucleation rate of zero, *a* = 1 for the constant nucleation, *a* > 1 for an increasing nucleation rate while 0 < *a* < 1 for a decreasing nucleation rate.^[^ ^36a^ ^]^ Parameter *b* represents the growth dimensionality and its value varies from 1, to 2, and to 3, corresponding to 1D, 2D, and 3D growth mechanisms, respectively. Parameter *c* represents the specific growth mode: 0.5 for the diffusion‐controlled growth and 1 for phase boundary (interface) controlled growth. The Avrami exponent *n* decreases with the increase of charging rates from ≈1.3 at 0.27 C to ≈0.5 at 4.4 C. Given the fitted values of *n* and available values of parameters *a*, *b*, and *c* in JMAK model, we propose *b* = 1 and *c* = 0.5 for the LiC_12_ → LiC_6_ transition, with *a* ranging from 0 to 1. These kinetic parameters are summarized in **Table** **1**. Particularly, the nucleation index *a* keeps decreasing from ≈0.8 at 0.27 C to almost 0 at 4.4 C, indicating nucleation rate decreases under increasing charging rates. Thus, with *b* = 1 indicating 1D growth and *c* = 0.5 of diffusion‐controlled growth, the result suggests that the LiC_12_ → LiC_6_ transition is kinetically controlled by diffusion, 1D growth process with decreasing nucleation rate under increasing charging rates.

(3) $$\mathrm{Li}{\;}^{+}+\mathrm{Li}C_{12}\;+\;e^{-}\to 2\mathrm{Li}C_{6}$$

**Table 1:** Kinetic parameters for the formation of LiC_6_ derived from NPD data under CC stage

Type	Exponent *n*	*a*	*R* ^2^
0.27 C	1.356(40)	0.856(40)	0.974
1.6 C	1.373(62)	0.873(62)	0.979
2.4 C	0.804(27)	0.304(27)	0.988
3.2 C	0.622(77)	0.122(77)	0.889
4.4 C	0.504(77)	0.004(77)	0.991

As shown in Equation (3), the LiC_12_ → LiC_6_ transition involves the following factors: (1) Li^+^ diffusing from liquid electrolyte to the edges of graphite flakes; (2) Li^+^ passing through the SEI layer; and (3) Li^+^ intercalation/diffusion within bulk graphite and corresponding charge compensation. For Li^+^ passing through the SEI layer, the studies by Dees et al. show that the interfacial impedance remains almost constant and there are no rate effects on this diffusion process up to10 C.^[^ ^39^ ^]^ In addition, graphite possesses good electronic conductivity in the order of 1.0 × 10^0^ to 2.45 × 10^5^ S cm^−1^, much higher than Li^+^ ionic conductivity within graphite.^[^ ^40^ ^]^ Lithiated graphite also exhibits higher electrical conductivity at higher degree of Li^+^ intercalation. Therefore, the influence of electronic conductivity on the formation of LiC_6_ is negligible. It is seen that the Li^+^ diffusion within lithiated graphite and liquid electrolyte are both involved for this transition. For Li^+^ diffusion within graphite/GICs, most experimental studies indicate a range from 10^−10^ to 10^−12^ cm^2^ s^−1^ at room temperature, due to various methods used and the difference (graphite type and microstructure) of graphite tested.^[^ ^41^ ^]^ The typical value for Li^+^ diffusion coefficient of liquid electrolyte is *D* = 4 × 10^−6^ cm^2^ s^−1^, much higher than the Li^+^ diffusion within graphite.^[^ ^3^, ^42^ ^]^ Moreover, both experiments and simulations suggest a decreasing Li^+^ diffusion with more Li^+^ intercalated, especially for the LiC_12_ → LiC_6_ transition (with 0.5 < x < 1 for Li*_x_* C_6_), due to the larger energy barrier for the diffusion with dense Li ions between graphene layers.^[^ ^25b^ ^]^ Thus, the relatively sluggish Li^+^ diffusion in the bulk graphite particles is the controlling factor for the LiC_12_ → LiC_6_ phase transition.

Besides this sluggish diffusivity of Li^+^, the highest Gibbs free energy barrier for LiC_12_ →LiC_6_ phase transition is another possible reason for the relatively slower LiC_12_ →LiC_6_ phase transition kinetics and the delay in phase transition under higher charging rates. This has been reported by others. Smith et al. verified this highest energy barrier for LiC_12_ →LiC_6_ transition via the multi‐layer phase field model with the Cahn–Hilliard reaction mechanism.^[^ ^37^ ^]^ In line with this theoretical result, Donal P. Finegan et al. found the switch of lithiation along depth profile under 2 C charging: initially, lithiation occurred dominatingly in the frontal region, then it shifted to deeper regions, and finally went back to the frontal region.^[^ ^15^ ^]^ This phenomenon indicates the high energy barrier for this LiC_12_ →LiC_6_ transition. The overpotential at the frontal region was initially less than that needed to overcome this barrier, thus lithiation occurred in deeper regions. Once this overpotential for the phase transition was satisfied, lithiation went back to the frontal regions. This is consistent with our current observations.

For nucleation rate parameter *a*, it is close to 1 at charging rates of 0.27 C and 1.6 C. This indicates that the nucleation rate for new LiC_6_ phase is almost constant when the charging rate is low and/or moderate, suggesting a smooth transition from LiC_12_ to LiC_6_. With the increase of charging rate to 2.4 C, parameter *a* decreases significantly to 0.304, which is due to insufficient Li ions available for the nucleation of new LiC_6_ phase. The reason can be easily explained by the large concentration gradient needed for the high charge rate (large current).^[^ ^43^ ^]^ With further increase of the charge rate (current), parameter *a* decreases greatly and reaches near 0 at 4.4 C, which indicates the nucleation of new LiC_6_ phase region is largely limited for fast charging.

**Figure 9:**
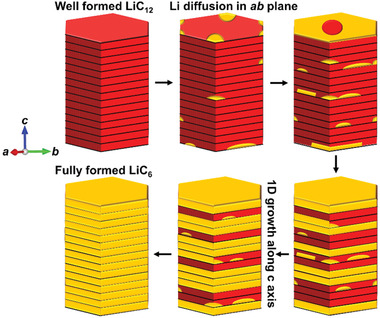
The 1D growth for the LiC_6_ along *c* direction of graphite flake.

In our study, Parameter *b* from the JMAK model suggests a 1D growth of nucleated LiC_6_, we attribute this 1D growth to the aggregation of LiC_6_ along the *c* direction of graphite flakes, as schematically shown in **Figure** **9**. As revealed by the Daumas–Herold domain model and following theoretic studies, the well‐formed LiC_12_ phase follows the AA(Li)AA··· stacking, and the space between every two neighboring graphene layers are available for further intercalation of Li^+^ to form denser LiC_6_ phase, as a response to reducing energy for the whole system.^[^ ^44^ ^]^ Both simulation and experiments indicate that it is impossible for intercalated Li^+^ to penetrate graphene layers along the *c* direction, given the large energy barrier of ≈10 eV, and intercalated Li^+^ only diffuses within *ab* plane of graphene layers.^[^ ^44^ ^]^ Considering the nonuniform morphology of graphite edges and the randomness of Li intercalation, Li^+^ intercalates into some graphene sheets earlier than other sheets. Meanwhile, intercalated Li^+^ exhibits intralayer attraction and interlayer repulsion, and the interlayer long‐ranged electrostatic repulsion is screened out only over certain layers of graphene along the *c* direction.^[^ ^26^, ^45^ ^]^ Thus, graphene layers intercalated by Li^+^ would hinder the intercalations of adjacent graphene sheets. Combined with the randomness of Li^+^ intercalation and the nonuniform morphology of graphite flakes, parallel Li^+^ intercalations between empty graphene layers simultaneously are hindered. Thus, we propose some graphene layers are fully intercalated earlier than other layers, with adjacent layers less lithiated or even without any Li^+^ intercalated, exhibiting a layer by layer intercalation behavior, to some extent. Following Li^+^ will repeat this serial intercalation process until the full formation of LiC_6_. Thus, the transition of LiC_12_ to LiC_6_ exhibits a 1D growth mechanism. A similar 1D growth behavior was also observed by Evans et al. during the intercalation of Li^+^ into layered TiS_2_.^[^ ^46^ ^]^ And similar “selective” deintercalation of H_2_SO_4_ from certain graphene layers was also observed for the opposite transition from stage I GIC to stage II GIC transition.^[^ ^47^ ^]^ Taken together, the monotonic decrease of *n* from the JMAK model with increased charging rates suggests 1D growth, diffusion‐controlled with decreasing nucleation rates for the transition of LiC_6_ from LiC_12_.

## 3 Conclusion

In this study, the fast operando neutron powder diffraction experiment is implemented via customized cylindrical cell. The good signal‐to‐noise ratio of collected operando data enables the multi‐phase Rietveld refinement and quantitative structural analysis of both NMC622 cathode and graphite anode. It is found that the structural evolution of NMC622 during the Li deintercalation process is consistently determined by its lithium contents up to 4.4 C charging rate, suggesting that NMC622 cathode is unlikely the limiting factor for fast charging of NMC/graphite cell. Instead, we found that the stage II (LiC_12_) → stage I (LiC_6_) transition during lithiation of graphite is the rate limiting step when charging the full cell to above 3 C. This is further confirmed by quantitatively study of the evolution of LiC_12_ and LiC_6_ using the JMAK model, where the Avrami exponent *n* decreases from ≈1.3 to ≈0.5 as the charging rate increases from 0.27 C to 4.4 C. This indicates that the stage II (LiC_12_) → stage I (LiC_6_) transition is a 1D diffusion controlled growth with decreasing nucleation kinetics under increasing charging rates. In all, this study sheds light on the comprehensive understanding and further optimization of rate performance of LIBs using graphite anode.

## Conflict of Interest

The authors declare no conflict of interest.

## Supporting information

Supporting Information

## Data Availability

Research data are not shared.
