# Supplementary material for: Structural Evolution and Transition Dynamics in Lithium Ion Battery under Fast Charging: An Operando Neutron Diffraction Investigation
Source: Adv Sci (Weinh). 2021 Sep 8;8(21):2102318. doi: 10.1002/advs.202102318 (PMC8564430; doi:10.1002/advs.202102318)
Supplement: Supplementary file 1 — Supporting Information [file ADVS-8-2102318-s001.pdf]

© 2021 Wiley-VCH GmbH

ADVANCED SCIENCE
Open Access

Supporting Information

for *Adv. Sci.,* DOI: 10.1002/advs.202102318

Structural evolution and transition dynamicsin Lithium ion battery under fast charging:an operando neutron diffraction investigation

Xianyang Wu, Bohang Song, Po-Hsiu Chien, S. Michelle Everett, Kejie Zhao,Jue Liu, Zhijia Du**

Support Information

Structural evolution and transition dynamicsin Lithium ion battery under fast charging:an operando neutron diffraction investigation

Xianyang Wu[a,b,c], Bohang Song[c], Po-Hsiu Chien[c], S. Michelle Everett[c], Kejie Zhao[b],Jue Liu[c,z],

Zhijia Du[a,z],

a Electrification and Energy Infrastructures Division, Oak Ridge National Laboratory, Oak Ridge, TN 37830, USA

b School of Mechanical Engineering, Purdue University, West Lafayette, IN 47907, USA

c Neutron Scattering Division, Oak Ridge National Laboratory, Oak Ridge, TN 37830, USA

-corresponding to Jue Liu (liuj1@ornl.gov)

-corresponding to Zhijia Du (duz1@ornl.gov, +1-(865)574-7519)

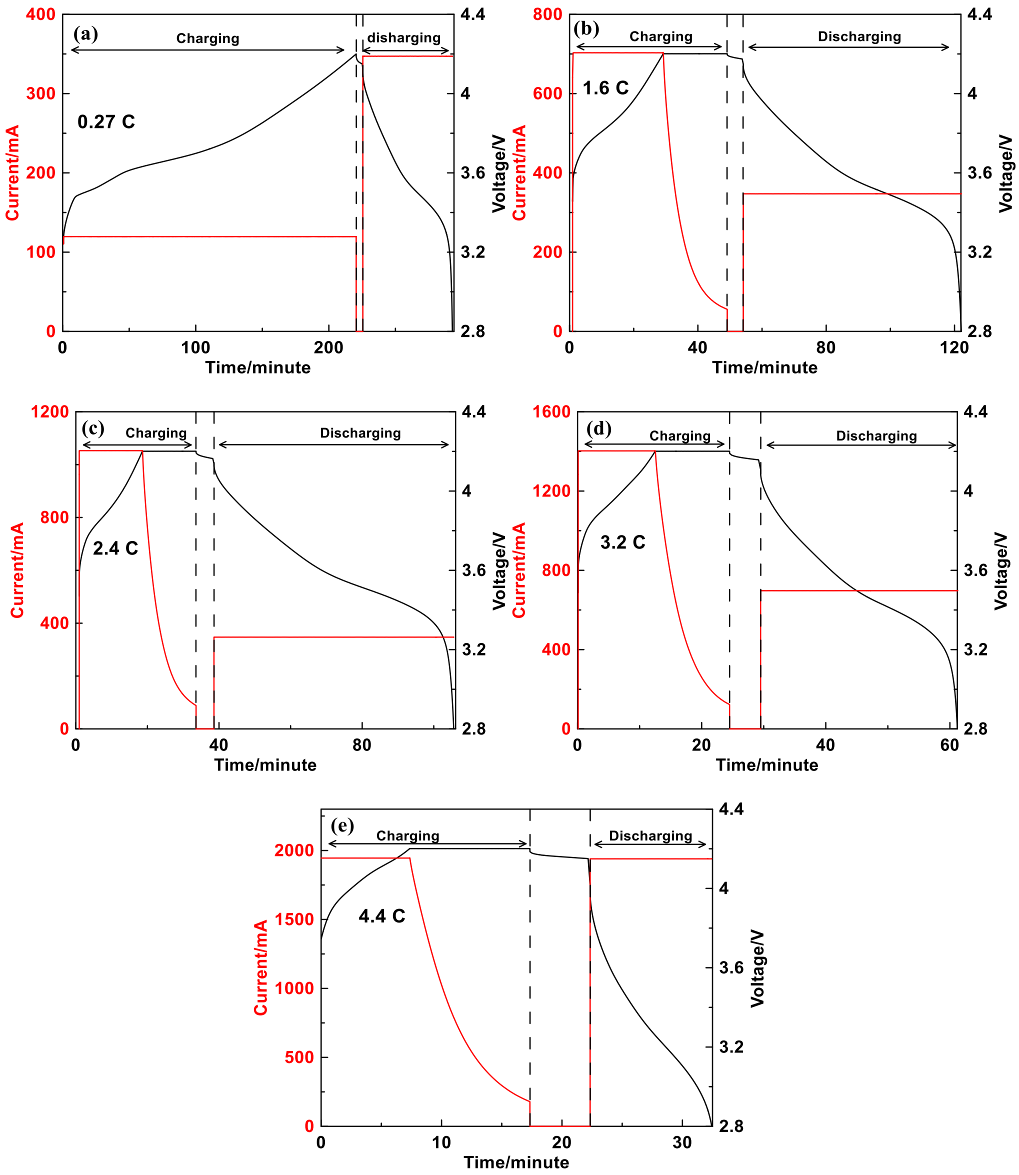

Figure S1.The detailed electrochemical performance of the cylindrical cell under 5 charging rates (a) Rate 1, 0.27 C; (b) Rate 2, 1.6 C; (c) Rate 3, 2.4 C; (d) Rate 4, 3.2 C; (e) Rate 5, 4.4 C.

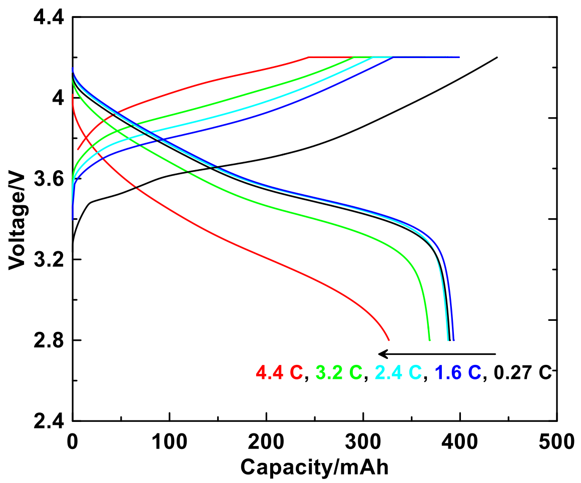

Figure S2. The voltage-capacity curve under all the 5 charging rates

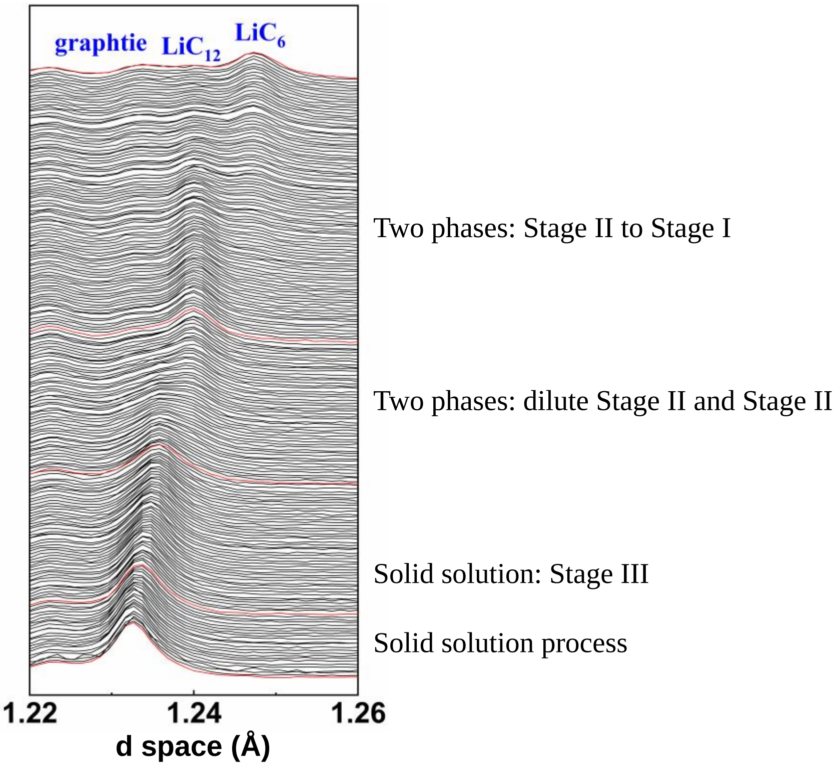

Figure S3. The phase transition of lithiated graphite during the 0.27 C charging

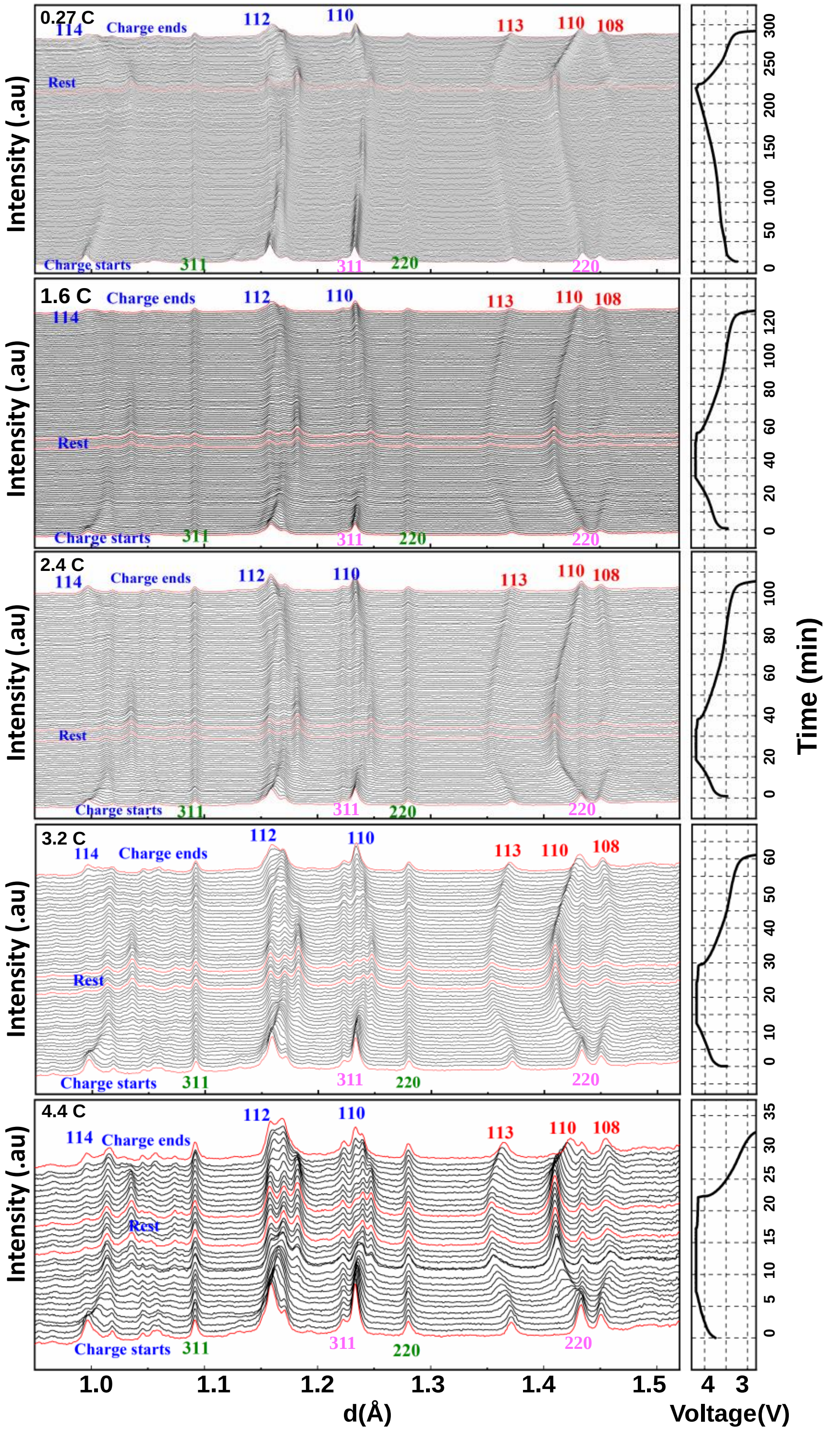

Figure S4. The diffraction patterns alongside with the voltage profile for all 5 rates, with major Bragg peaks from lithiated graphite, NMC622, Cu, Al current collector labelled in blue, red, olive and magenta.

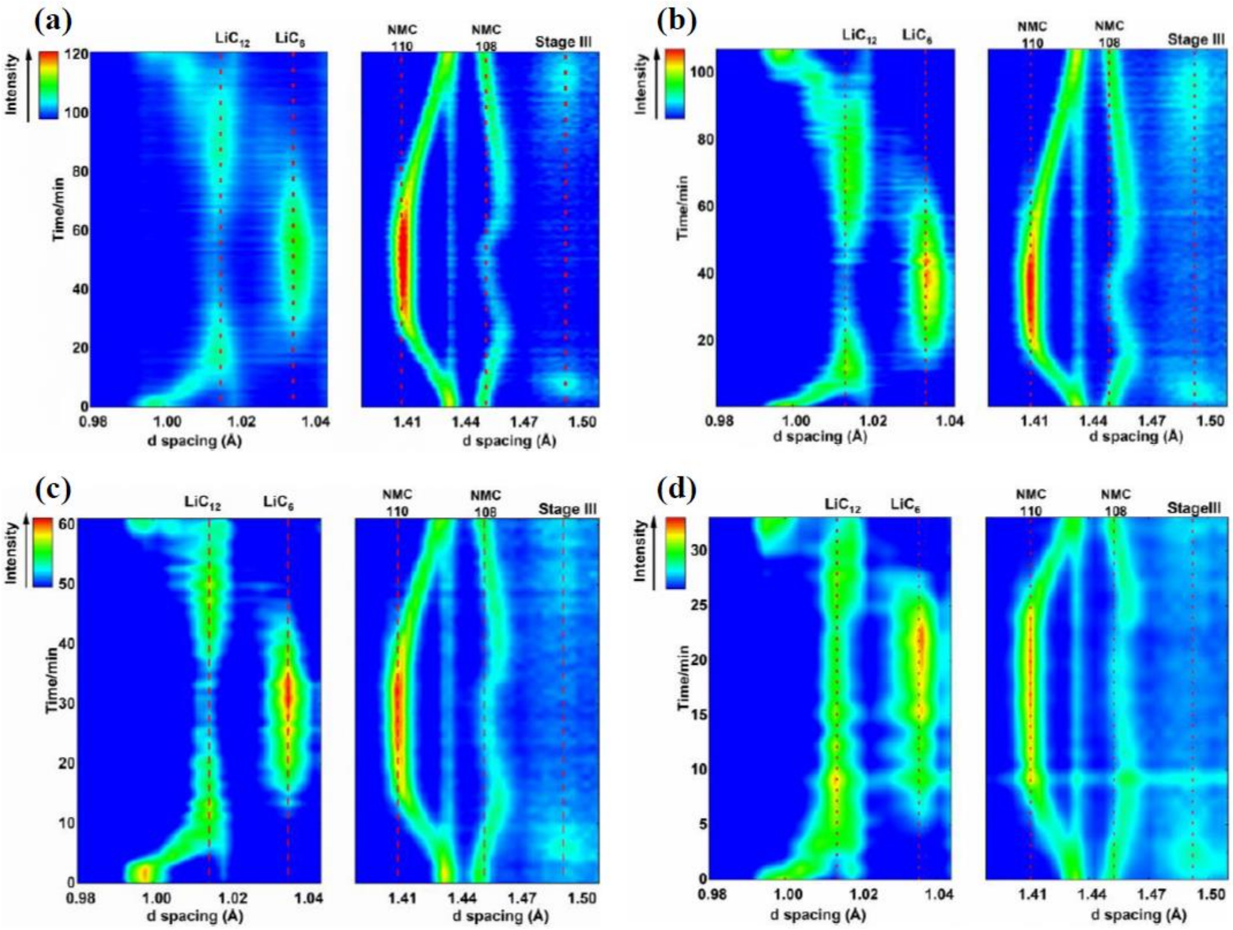

Figure S5. The phase evolution of Stage III, Stage II (LiC_{12}), Stage I(LiC_6) under higher charging rates: (a) 1.6 C; (b) 2.4C; (c) 3.2 C; (d) 4.4 C.

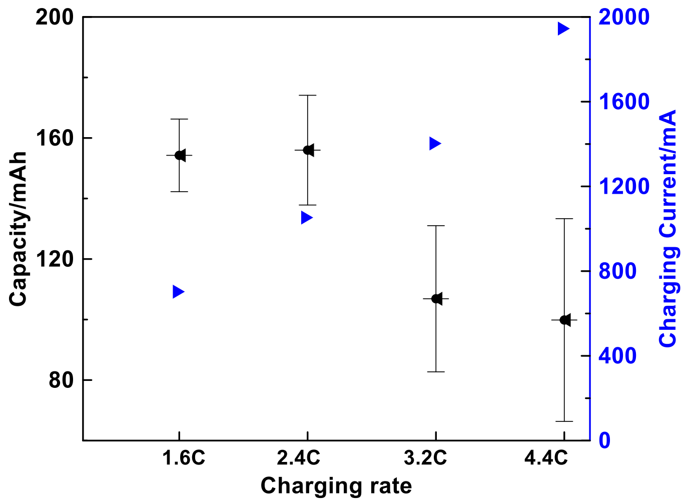

Figure S6. The capacity for the first show-up of stage I (LiC_6) within the cell under charging rates from 1.6 C to 4.4C

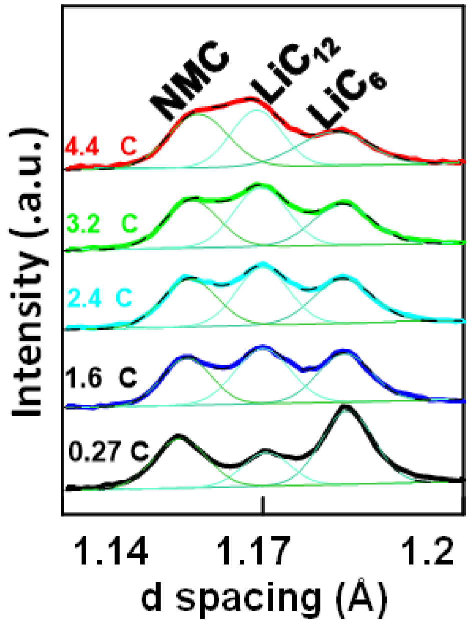

Figure S7. The qualitative comparison of LiC_6 and LiC_{12} contents under the 5 charging rates, the black dot line represents the cumulative line of all these 3 fitted peaks, the spring green line is fitted NMC peak, the turquoise line is fitted LiC_{12} peak and the sea green is fitted LiC_6 peak.

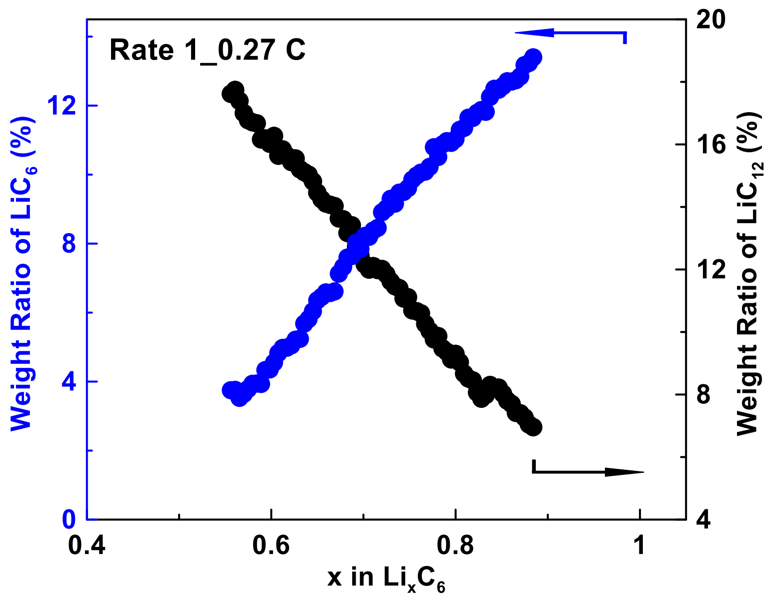

Figure S8.The time evolution of LiC_{12} and $LiC_6$for the whole charging process under 0.27 C.
